# The Effect of Sodium Alginate and Pectin Added to a Carbohydrate Beverage on Endurance Performance, Substrate Oxidation and Blood Glucose Concentration: A Systematic Review and Meta-analysis

**DOI:** 10.1186/s40798-022-00472-5

**Published:** 2022-06-21

**Authors:** Shaun Sutehall, Borja Muniz-Pardos, Andrew Bosch, Yannis Pitsiladis

**Affiliations:** 1 Division of Physiological Sciences, Department of Human Biology, University of Cape Town, Cape Town, South Africa; 2 GENUD (Growth, Exercise, Nutrition and Development) Research Group, University of Zaragoza, Zaragoza, Spain; 3 School of Sport and Health Sciences, University of Brighton, Welkin House, 30 Carlisle Road, Eastbourne, BN20 7SN UK

**Keywords:** Hydrogel, Carbohydrate, Endurance, Exercise, Systematic, Meta-analysis, Performance

## Abstract

**Introduction:**

Scientific and public interest in the potential ergogenic effects of sodium alginate added to a carbohydrate (CHO) beverage has increased in the last ~ 5 years. Despite an extensive use of this technology by elite athletes and recent research into the potential effects, there has been no meta-analysis to objectively elucidate the effects of adding sodium alginate to a CHO beverage on parameters relevant to exercise performance and to highlight gaps in the literature.

**Methods:**

Three literature databases were systematically searched for studies investigating the effects of sodium alginate added to CHO beverage during prolonged, endurance exercise in healthy athletes. For the systematic review, the PROSPERO guidelines were followed, and risk assessment was made using the Cochrane collaboration’s tool for assessing the risk of bias. Additionally, a random-effects meta-analysis model was used to determine the standardised mean difference between a CHO beverage containing sodium alginate and an isocaloric control for performance, whole-body CHO oxidation and blood glucose concentration.

**Results:**

Ten studies were reviewed systematically, of which seven were included within the meta-analysis. For each variable, there was homogeneity between studies for performance (*n* = 5 studies; *I*^2^ = 0%), CHO oxidation (*n* = 7 studies; *I*^2^ = 0%) and blood glucose concentration (*n* = 7 studies; *I*^2^ = 0%). When compared with an isocaloric control, the meta-analysis demonstrated that there is no difference in performance (*Z* = 0.54, *p* = 0.59), CHO oxidation (*Z* = 0.34, *p* = 0.71) and blood glucose concentration (*Z* = 0.44, *p* = 0.66) when ingesting a CHO beverage containing sodium alginate. The systematic review revealed that several of the included studies did not use sufficient exercise intensity to elicit significant gastrointestinal disturbances or demonstrate any ergogenic benefit of CHO ingestion. Risk of bias was generally low across the included studies.

**Conclusions:**

This systematic review and meta-analysis demonstrate that the current literature indicates no benefit of adding sodium alginate to a CHO beverage during exercise. Further research is required, however, before firm conclusions are drawn considering the range of exercise intensities, feeding rates and the apparent lack of benefit of CHO reported in the current literature investigating sodium alginate.

## Key Points

- The current empirical evidence indicates no benefit of adding sodium alginate to a carbohydrate beverage.
- Much of the current literature does not use sufficiently high carbohydrate intake or exercise intensity to elicit gastrointestinal distress in the control conditions, and thus, the effect of sodium alginate on gastrointestinal comfort is not well studied.

## Introduction

While it is now widely recognised that ingestion of carbohydrate (CHO) during prolonged exercise improves endurance performance [1], the situation as recent as the 1970s was very different with studies such as by Costill et al. [2] questioning the value of drinking during marathon competition. This conclusion was made despite a better maintained blood glucose concentration and respiratory exchange ratio with the ingestion of a glucose–electrolyte solution during a two-hour treadmill run, compared with water. Since then, the number of studies investigating CHO ingestion has soared, with published studies containing the words “exercise AND carbohydrate” increasing from 61 in 1970, to 1146 in 2020 (PubMed search). Current consensus suggests that athletes should ingest CHO at 30–60 g hr^−1^ during exercises lasting 1–2.5 h and up to 90 g hr^−1^ during exercise lasting longer than 2.5 h [3]. Significant research has been performed with the aim of identifying the maximal rate at which exogenous CHO (ExCHO) is oxidised [4–7] with ~ 1.75 g min−1 as the highest reported within the literature [4]. To achieve these high rates of ExCHO, participants ingested a mixture of glucose and fructose at 2.4 g min^−1^, far exceeding the recommended 1.5 g min^−1^ and also significantly higher than what has been reported to be ingested by athletes during a competitive marathon [8]. This disparity between recommendations and observed habits of athletes may be, in part, due to the increasing prevalence of gastrointestinal distress (GID) with increasing hypotonicity of CHO beverages [9], preventing athletes from ingesting CHO at rates recommended in the literature.

Recently, a new development in CHO provision has emerged (i.e. hydrogel technology) and may have the potential to improve the effectiveness of CHO ingestion during exercise [10]. The theoretical mechanisms behind the use of hydrogels is that CHO beverages containing sodium alginate and pectin form a pH-sensitive hydrogel, encapsulating the ingested CHO once the beverage comes in contact with the acidic stomach environment. This then enters the small intestine, where the hydrogel subsequently dissipates and releases the CHO for absorption in accordance with a rise in intestinal pH [10]. Evidence for these mechanisms has been published, firstly via magnetic resonance imaging (MRI) of participants’ stomachs after ingesting a CHO beverage containing sodium alginate and pectin, and demonstrating that a viscous gel was formed within the stomach [11]. Secondly, it has been witnessed that the early gastric emptying (GE) rate of subjects ingesting a CHO beverage at rest is enhanced with the addition of sodium alginate and pectin [12], suggesting that the sensing of the ingested CHO by the receptors controlling the rate of GE is altered by the hydrogel. Further research is required to replicate this finding of enhanced early GE rate considering the range of studies demonstrating a slowing [13, 14] or no effect [15] when ingesting test meals/beverages containing sodium alginate and/or pectin in various forms and ingestion methods. Consuming beverages containing CHO and sodium alginate has become widely adopted by elite athletes, with claims of reduced GID associated with its use in competition [16].

While there is yet to be any studies specifically aimed at investigating the potential GID reducing effects, a growing number of studies have been performed with the aim to identify any potential ergogenic effects associated with CHO ingestion with sodium alginate and pectin. Two narrative reviews have emerged discussing the potential efficacy and effectiveness of this development [17, 18], although both relied on non-systematic approaches to review the literature. While this may be useful for interpreting the literature, a more reliable and accurate method, using quantitative data where appropriate, should be used to make conclusions on the published literature. Employing a systematic review and meta-analysis may also highlight any trends observed within the data in addition to assessing the limitations of the current literature and provide new potential directions for future research.

Therefore, the aim of this systematic review and meta-analysis is to examine the potential effects of adding sodium alginate and pectin on athletic performance, substrate oxidation and blood glucose concentration in healthy athletes.

## Methods

The current systematic review and meta-analysis was conducted in accordance with the Cochrane handbook for systematic reviews of interventions [19] and following the recommendations of the Preferred Reporting Items for Systematic Reviews and Meta-Analyses (PRISMA) [20].

### Data Sources and Search Strategy

**Fig. 1:**
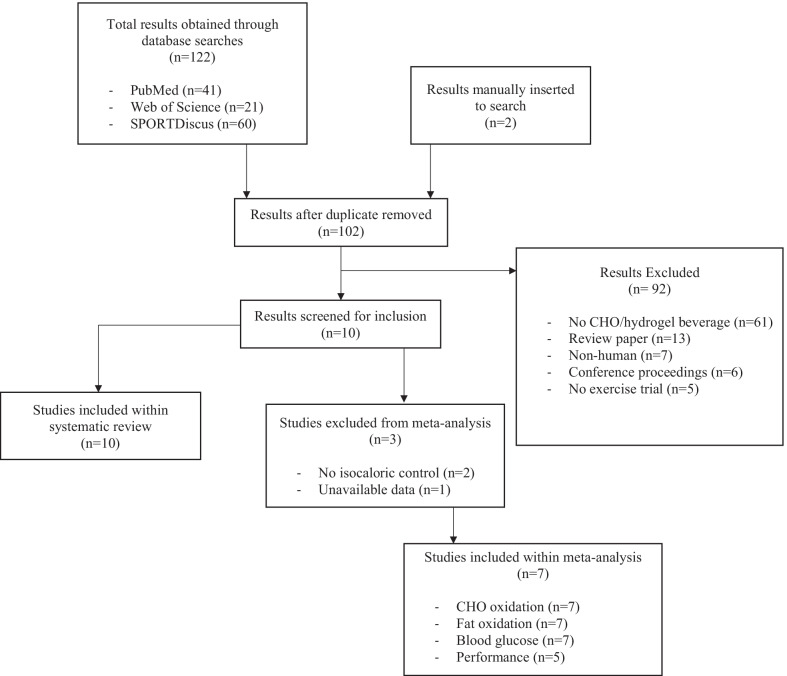
Flow chart of the systematic and meta-analysis process

The databases PubMed, Web of Science and SPORTDiscus were used to search for eligible studies to be included for this systematic review and meta-analysis. The first search was performed in November 2020 and updated in August 2021. The search was limited to English language, and there were no restrictions regarding the year of publication. The specific search terms were PubMed: (“carbohydrates” [Mesh] OR “carbohydrate”) AND (“exercise” [Mesh] OR “exercise” OR “exercise Test” [Mesh] OR “exercise tolerance” [Mesh]) AND (“hydrogels” [Mesh] OR “hydrogel” OR “alginates” [Mesh] OR “pectin”), Web of Science: (ALL = (carbohydrate) OR ALL = (carbohydrates)) AND (ALL = (exercise) OR ALL = (exercise test) OR ALL = (exercise tolerance)) AND (ALL = (hydrogel) OR ALL = (hydrogels) OR ALL = (alginate) OR ALL = (pectin)) and SPORTDiscus: (TX carbohydrates OR TX carbohydrate) AND (TX exercise OR TX exercise test OR TX exercise tolerance) AND (TX hydrogels OR TX hydrogel OR TX alginates OR TX pectin). The reference list of each study identified was scrutinised for any further studies to be included within the analysis. The strategic search and study selection were performed independently by two researchers (SS and BMP), with any discrepancies in included studies resolved by consultation of AB. The number of studies was identified, and their selection process is presented in Fig. 1.

### Eligibility Criteria

The inclusion criteria for the meta-analysis focussed on the population characteristics, on the study design, on the exercise test, on the beverage characteristics, and on the main outcomes: 1) population: male or female athletes, between 18 and 50 years old, 2) study design: randomised allocation/order in a crossover design, using a control beverage, 3) exercise test: a bout of prolonged endurance exercise of at least 60 min (i.e. running, cycling, rowing, skiing and swimming), and 4) beverage characteristics: subjects ingesting a CHO beverage with sodium alginate and an isocaloric control beverage containing the same composition of CHO without sodium alginate. These athletes had to be healthy, with no history or presently showing symptoms of gastrointestinal disease.

Those studies that failed to meet the criteria for meta-analysis, but included CHO beverage ingestion with sodium alginate and a prolonged endurance exercise with measurements of physiological variables (i.e. CHO oxidation, blood glucose concentration), were included within the systematic review.

### Study Selection and Data Collection

After an initial screening and removal of duplicates, the titles and abstracts were assessed for suitability. The full texts of the remaining studies were examined, and a final list of studies meeting the inclusion criteria created. The following data were extracted from these selected studies: (1) title, type of publication (original, review, letter, conference proceedings), information on publication (authors, year, research centre of department); and (2) participant characteristics, study design, control group (randomisation and beverage composition), experimental group (beverage composition and presence of sodium alginate and pectin) and outcomes (performance, total CHO oxidation, fat oxidation and blood glucose concentration).

Data extraction for the meta-analysis was collated, primarily through text and tables, and if the data were present in a figure only, the graph was digitised using an in-built measuring tool (Adobe Acrobat Reader DC, Adobe Inc., California, USA) with the means and SD measured manually, using the scale provided in the figure. In studies where data were reported across numerous time points, data were averaged to calculate a single mean and SD. Substrate oxidation is presented as g min^−1^ and blood glucose concentration as mMol L^−1^. Blood glucose concentration data that were presented as mg dL^−1^ were converted to mMol L^−1^ by multiplying the value by 0.0555. If data could not be extracted through the text, tables or figures, the corresponding author was contacted.

### Assessment of Risk of Bias

Independently, two researchers (SS and BMP) used the Cochrane Collaboration’s tool for assessing the risk of bias [21] and determined the risk of bias in each study. Each of the following categories was assessed as either being at high risk, low risk or unclear risk of bias: random sequence generation, blinding of participants and personnel, blinding of outcome assessment, incomplete outcome data and selective reporting. Any discrepancies were discussed between SS and BMP with any remaining disagreements resolved by AB.

### Statistical Analysis

Review Manager (RevMan, version 5.4.1, The Cochrane Collaboration, 2020 [22]) was used for the meta-analysis. Outcome measures for total CHO oxidation, fat oxidation and blood glucose concentration were quantified between groups using the mean difference (MD) with 95% confidence intervals (CIs). Due to the different methods used that assessed the performance, this outcome measure was calculated using the standardised mean difference (SMD) with 95% CI. A random-effects model was used to determine the effect size for all outcome measures. The heterogeneity of the effect between studies of the treatment effect was assessed through the *I*^2^ statistic and the chi-square test [23]. The MD and SMD were interpreted as a small effect (0.2–0.4), moderate effect (0.5–0.7) and large effect (> 0.8). Significance was set at *p* ≤ 0.05.

## Results

### Search Results and Study Characteristics

The flow chart presented in Fig. 1 details the process used to compile the eligible studies for inclusion into the present systematic review and meta-analysis. Following an initial search, 41, 21 and 60 studies were identified from PubMed, Web of Science and SPORTDiscus, respectively. Additional two studies were manually entered. After removal of duplicates, a total of 102 studies were considered for this initial stage. Following screening of abstracts, the number of articles was reduced to 10 manuscripts, which were carefully read, and, when eligibility criteria was applied, 10 and 7 studies were finally included for the systematic review and meta-analysis, respectively. The characteristics of these studies are summarised in Table 1.

**Table 1:** Studies included within meta-analysis and systematic review

Authors	Participants (training status)	Type of activity during the trials	Performance measure	Experimental beverage	Control beverage	Primary outcomes	Results
Rowe et al. 2021 [31]*	11 M (well-trained)	120 min treadmill running at 68%${\dot{\text{V}}\text{O}}_{2} \max$	5 km time trial	1.5 g min^−1^ maltodextrin + fructose + SA + P	1.5 g min^−1^ maltodextrin + fructose	Performance, substrate oxidation, blood metabolites	Sig faster time trial and higher CHO oxidation No difference in plasma glucose concentration, lactate or NEFAs
Pettersson et al. 2020 [27]	12 (sex not reported, endurance-trained)	180 min cycling at 55% Wmax	None	1.6 g min^−1^ maltodextrin + fructose + SA + P	(1) 1.6 g min^−1^ Vitargo Pure® + Vitargo electrolyte® (starch + electrolytes) + amylopectin (2) 1.6 g min^−1^ Isostar Endurance Plus® (Maltodextrin + sucrose)	Oral health, ExCHO oxidation, substrate oxidation, blood metabolites	Sig higher ExCHO oxidation with experimental beverage Sig lower blood glucose concentration 40 min after cessation of CHO feeding with experimental beverage Attenuated decline in teeth pH
McCubbin et al. 2020 [24]*	9 M (recreational and elite)	180 min cycling at 60%${\dot{\text{V}}\text{O}}_{2} \max$	Time to exhaustion	1.5 g min^−1^ maltodextrin + fructose + SA + P	1.5 g min^−1^ maltodextrin + fructose	Performance, substrate oxidation, blood metabolites	No sig differences
Flood et al. 2020 [29]*	7 M, 7 F (recreational)	90 min cycling at 45% ${\dot{\text{V}}\text{O}}_{2} \max$ in 32 °C and 70% relative humidity	Work completed in 15 min	1.5 g min^−1^ maltodextrin + fructose + SA + P	1.5 g min^−1^ maltodextrin + fructose	Performance, substrate oxidation, blood metabolites, intestinal integrity	No sig difference in performance, substrate oxidation, performance or intestinal integrity Greater gut discomfort with experimental beverage
Myrick et al. 2019 [32]	1 (sex and training status not reported)	Treadmill running at 12 min mile^−1^ until exhaustion	Time to exhaustion	Microgel dispersion + 39 g glucose	Commercial sports drink containing 39 dextrose	AUC blood glucose absorption kinetics	Higher AUC glucose absorption kinetics with microgel dispersion
Mears et al. 2019 [25]*	8 M (well-trained)	120 min cycling at 55% WMax	Time to complete set work	1.1 g min^−1^ maltodextrin + fructose + SA + P	1.1 g min^−1^ maltodextrin + fructose	Performance, substrate oxidation, blood metabolites	No sig difference in performance Sig higher RPE and stomach fullness with experimental beverage
Barber et al. 2019 [28]*	9 M (trained)	120 min treadmill running at 60%${\dot{\text{V}}\text{O}}_{2} \max$	None	1.5 g min^−1^ maltodextrin + fructose + SA + P	1.5 g min^−1^ maltodextrin + fructose	ExCHO oxidation, substrate oxidation, blood metabolites	No sig differences
Pettersson et al. 2019 [33]	12 (6 M, 6 F, trained)	120 min treadmill skiing 69% ${\dot{\text{V}}\text{O}}_{2} \max$ in − 5 °C	2400 m (males) or 2000 m (females) time trial	2.2 g min^−1^ maltodextrin + fructose + SA + P	Non-caloric placebo (sweetened water)	Performance, ExCHO oxidation, substrate oxidation, blood metabolites	Sig higher ExCHO, total CHO oxidation and sig lower fat oxidation with experimental beverage. No sig diff in performance
Baur et al. 2019 [26]*	9 M (trained)	98 min varied intensity cycling	10 maximal sprints	1.3 g min^−1^ maltodextrin + fructose + SA + P	1.3 g min^−1^ maltodextrin + fructose	Performance, substrate ox, blood metabolites	No sig differences
Sutehall et al. [30]*	8 M (well-trained)	105 min treadmill running 70%${\dot{\text{V}}\text{O}}_{2} \max$	None	1.2 g min^−1^ maltodextrin + fructose + SA + P	(1) 1.2 g min^−1^ maltodextrin + fructose (2) Water	ExGluc oxidation, substrate oxidation, blood metabolites	No sig differences

M, male; F, female; ${\dot{\text{V}}\text{O}}_{2} \max$, maximal oxygen uptake; %WMax, percentage of max Watts; SA, sodium alginate; P, pectin; ExCHO oxidation, exogenous carbohydrate oxidation rate; ExGluc oxidation, exogenous glucose oxidation rate; AUC, area under the curve

*Included within meta-analysis

Of the 10 studies included within the systematic review, four used cycling [24–27], five used running [28–32] and one used skiing as the mode of exercise [33]. The performance measures included time to exhaustion tests [24, 32], time trial tests [25, 29, 31, 33] and repeated sprint ability tests [26]. Five of the 10 articles included in the systematic review were included in the meta-analysis for performance and seven for total CHO oxidation and blood glucose concentration. Two studies were discarded from the meta-analytical study of performance as they did not include an isocaloric control beverage [27, 33] and one study did not provide sufficient data to calculate the required variables [32]. Therefore, 68, 61 and 50 participants were included for total CHO oxidation, blood glucose concentration and performance, respectively.

### General Systematic Review Results

#### Participant Characteristics

A total of 92 participants were included across the 10 studies included for the systematic review (79 males and 13 females). No SD data were provided in two studies [26, 32], and therefore the participant characteristics presented represent 8 of the 10 included studies (males: age 29 ± 4 yrs, body mass 73.8 ± 7.1 kg, maximal oxygen uptake (${\dot{\text{V}}\text{O}}_{2} \max$) 62.5 ± 5.8 mL kg min^−1^; and females: age 24 ± 4 yrs, body mass 64.8 ± 7.3 kg, ${\dot{\text{V}}\text{O}}_{2} \max$ 57.1 ± 7.5 mL kg min^−1^). Other participant variables were not uniformly reported across studies and therefore not reported.

#### Exercise Characteristics

Of the 10 included studies, six involved prolonged, continuous treadmill running for an average of 129 min (range 105—180 min) at an average intensity of 61% ${\dot{\text{V}}\text{O}}_{2} \max$ (range 45–71% ${\dot{\text{V}}\text{O}}_{2} \max$) [24, 28–32]. Four studies utilised a cycling protocol, from 90 to 180 min [25–27, 29]. In three of these studies, the cycling was continuous at 60% ${\dot{\text{V}}\text{O}}_{2} \max$ [27], 55% maximal Watts (Wmax, [25]) and 45% ${\dot{\text{V}}\text{O}}_{2} \max$ [29] and in one, an intermittent sprint protocol was used, with exercise intensity ranging from 30 to 80% Wmax [26]. Finally, one study utilised a continuous skiing protocol, lasting for 120 min performed at 70% ${\dot{\text{V}}\text{O}}_{2} \max$ [33].

#### Beverage Composition

Eight of the 10 included studies provided CHO as a combination of maltodextrin and fructose [24–30, 33]. One study provided the ingested CHO as glucose and fructose [31] and another as glucose only [32]. Most of the eight studies providing two CHO types used a ratio of 1:0.7 of maltodextrin/glucose:fructose, with the exception of the two studies of Pettersson et al. (1:0.8, [27, 33]), Baur et al. (2:1, [26]) and Rowe et al. (2:1, [31]). Of those eight studies that provided CHO as a combination of maltodextrin and fructose, four provided CHO at a rate of ~ 90 g hr^−1^ ([24, 27–29]). Notably, the ingestion rate of one of these four studies was 95 g hr^−1^ [27]; exercise continued after CHO ingestion was ceased, giving an “effective” ingestion rate of 65 g hr^−1^. Two studies provided CHO at ~ 70 g hr^−1^ [25, 30], one at 78 g hr^−1^ [26] and another at 132 g hr^−1^ [33]. A single study provided the CHO as glucose:fructose at a rate of 90 g hr^−1^ [31], and another study provided a single bolus of 39 g of glucose and thus an ingestion rate of 15 g hr^−1^ [32].

Nine studies included sodium alginate and pectin, with only one study using additional ingredients to alter the formation of a hydrogel [32]. Specifically, this study by Myrick et al. [32] formed cross-linked hydroxypropyl cellulose microparticles to which glucose powder and sodium alginate were added and mixed. Therefore, caution should be applied when comparing this study with others in which beverages only contained sodium alginate and pectin.

#### Meta-analysis Results

**Fig. 2:**
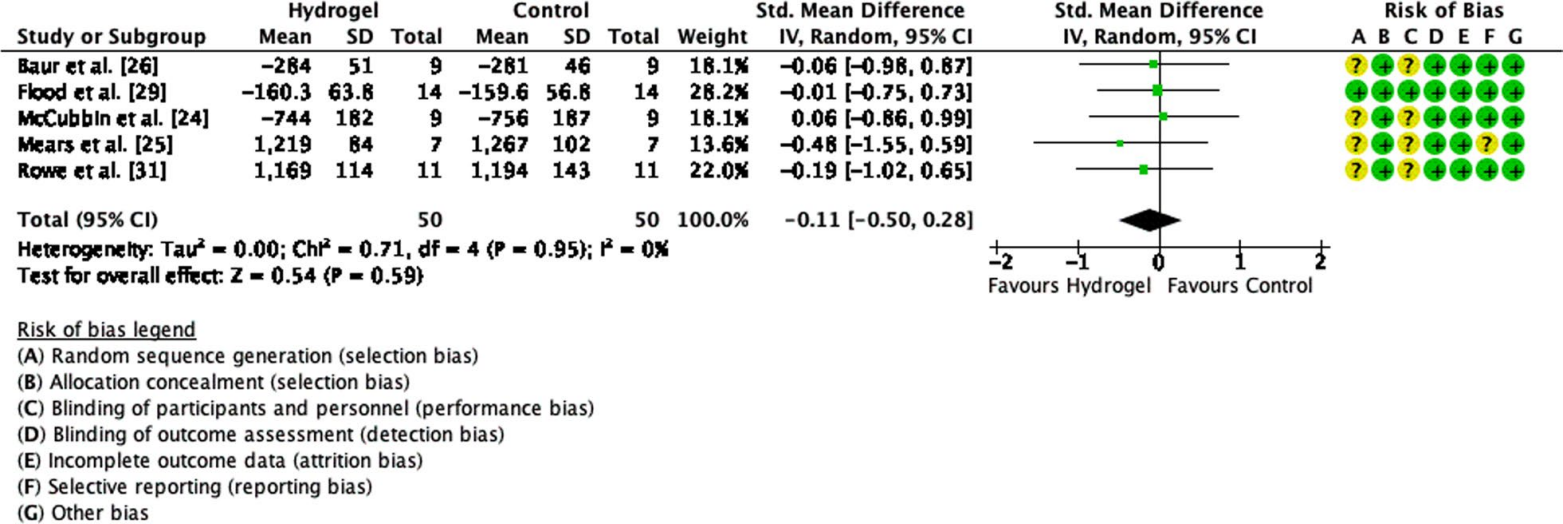
Forest plot comparing exercise performance between CHO beverage with sodium alginate and isocaloric control beverage. *SD* standard deviation, *std* standardised, *IV* inverse variance, *CI* confidence interval

**Fig. 3:**
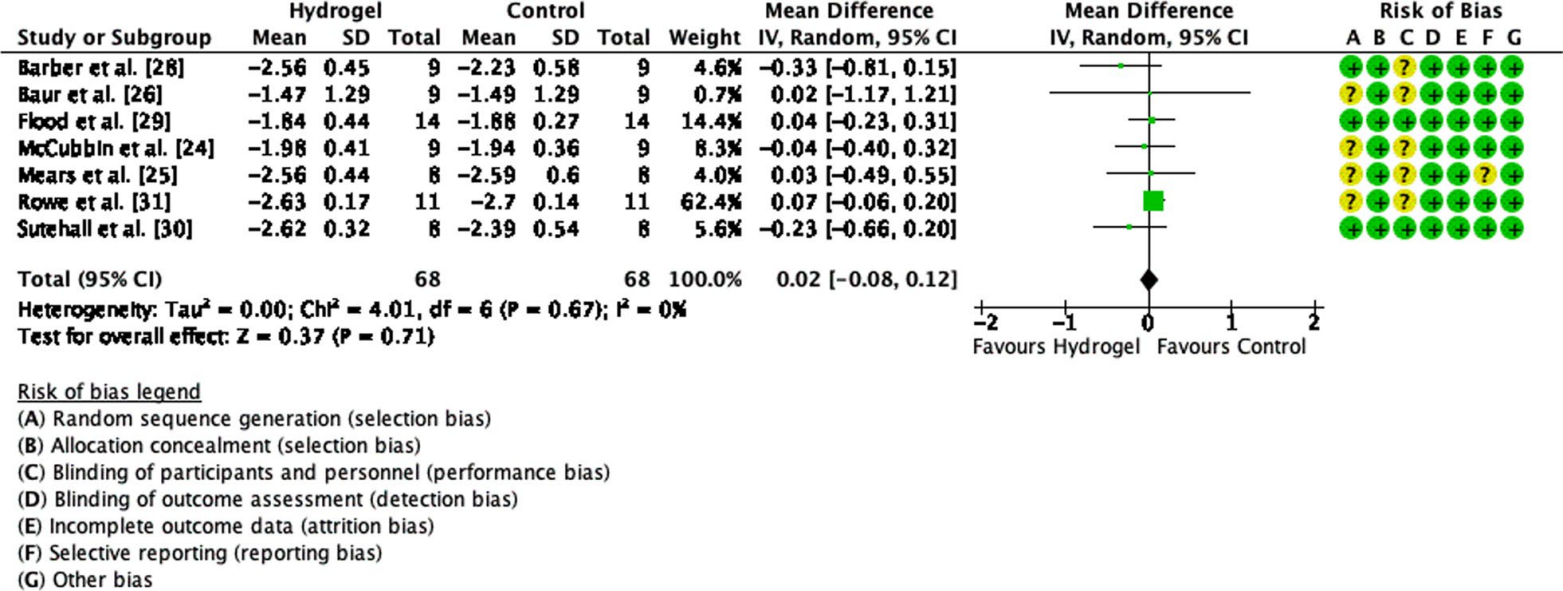
Forest plot comparing whole-body CHO oxidation rate between CHO beverage with sodium alginate and isocaloric control beverage. *SD* standard deviation, *std* standardised, *IV* inverse variance, *CI* confidence interval

**Fig. 4:**
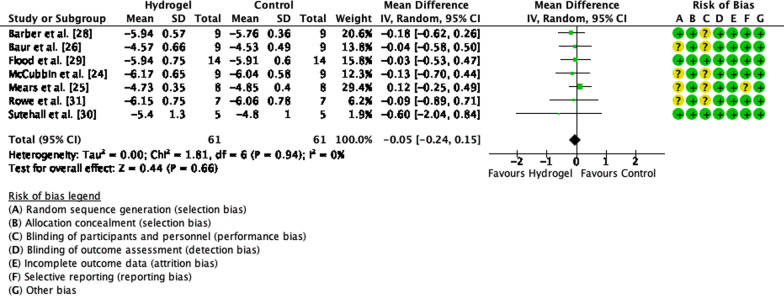
Forest plot comparing blood glucose concentration between CHO beverage with sodium alginate and isocaloric control beverage. *SD* standard deviation, *std* standardised, *IV* inverse variance, *CI* confidence interval

Five of the 10 articles included in the systematic review were further included in the meta-analysis for performance, seven for total CHO oxidation, seven for fat oxidation, and seven for blood glucose concentration. When assessing performance, a low heterogeneity was observed between studies (Tau^2^ = 0.0%, Chi^2^ = 0.71, *df* = 4 and *I*^2^ = 0%) and no difference in performance between CHO beverages with or without sodium alginate and pectin (*Z* = 0.54, *p* = 0.59, SMD =  − 0.11, 95% CI =  − 0.50, 0.28, Fig. 2). Similarly, there was low heterogeneity observed for CHO oxidation rate between studies (Tau^2^ = 0.0%, Chi^2^ = 4.01, *df* = 6 and *I*^2^ = 0%) and no differences in CHO oxidation rate when ingesting a CHO beverage with or without sodium alginate and pectin (*Z* = 0.37, *p* = 0.71, MD = 0.02, 95% CI =  − 0.08, 0.12, Fig. 3). Lastly, there were no differences in blood glucose concentration (*Z* = 0.44, *p* = 0.66, MD =  − 0.05, 95% CI =  − 0.24, 0.15) and low heterogeneity (Tau^2^ = 0.0%, Chi^2^ = 1.81, *df* = 6 and *I*^2^ = 0%, Fig. 4).

#### Risk of Bias

Due to incomplete reporting of random sequence in four of the seven studies included within the meta-analysis, it was unclear to what extent a selection bias occurred [24–26, 31]. Similarly, in five of the seven studies, the methods used to blind the participants/investigators were not well reported (i.e. reporting “…in this double-blind study…”) and resulted in an unclear performance bias [24–26, 28, 31]. One study did not fully report all data gathered [25].

## Discussion

The present systematic review and meta-analysis has reviewed all the relevant literature investigating the use of CHO beverages with additional sodium alginate and pectin during exercise. It was found that, based on published studies, the addition of sodium alginate and pectin confers no significant benefit compared to an isocaloric beverage on performance, CHO oxidation or blood/plasma glucose. Each comparison revealed excellent homogeneity of results (i.e. *I*^2^ = 0.0%), suggesting that the current meta-analysis provided an accurate quantitative representation of the effects of sodium alginate and pectin added to CHO beverages.

### Performance

The addition of sodium alginate and pectin to CHO beverages has become a popular trend in athletic competition since its public release in 2016 [10]. While the products containing sodium alginate and pectin have been widely used by athletes winning international races [34], the empirical evidence to date is less clear. Five studies have investigated the effect sodium alginate and pectin has on performance [24–26, 29, 31] and when assessed together, it was found that there was no difference in performance when participants ingested CHO with or without sodium alginate and pectin (*Z* = 0.54, *p* = 0.67, Fig. 2). The proposed mechanism through which additional sodium alginate and pectin may exert a beneficial effect during exercise is primarily through “hiding” the ingested CHO within a pH-sensitive hydrogel [10, 12], reducing the negative side effects of ingesting concentrated CHO beverages (e.g. GID). This theory has yet to be experimentally proven, and it remains unclear if this “hiding” mechanism is beneficial to performance. Recently, a study by Rowe et al. [31] has demonstrated a significant improvement in 5 km time trial performance following 120-min prolonged running at 68% ${\dot{\text{V}}\text{O}}_{2} \max$ while ingesting a CHO beverage containing sodium alginate and pectin in comparison with an isocaloric control beverage. It was also observed that the ingestion of the CHO beverage containing sodium alginate and pectin reduced upper and lower GID symptoms and increased the exogenous CHO oxidation rate, which the authors speculate are the mechanisms through which performance was improved [31].

CHO ingestion is most effective at improving performance in prolonged endurance events at relatively high intensities, where the contribution of CHO oxidation to total energy expenditure is highest. Five of the seven studies included within the meta-analysis comparing prolonged (90–180 min) endurance exercise used a relatively low intensity (~ 60% ${\dot{\text{V}}\text{O}}_{2} \max$) [24–26, 28, 29]. This exercise intensity is far lower than the ~ 80–85% ${\dot{\text{V}}\text{O}}_{2} \max$ sustained by elite athletes during marathons [35] and must be considered when interpreting the results. The effect of utilising this low intensity on performance assessments is highlighted but not discussed in a review by King et al. ([17] Fig. 3). The current studies assessing the potential performance implications of adding sodium alginate to a CHO beverage have used a wide range of exercise intensities during a “preload”, from relatively low (i.e. ≤ 65% ${\dot{\text{V}}\text{O}}_{2} \max$; [24, 25, 29]) to moderate (i.e. 68% ${\dot{\text{V}}\text{O}}_{2} \max$; [31]) to varied (i.e. ~ 60% ${\dot{\text{V}}\text{O}}_{2} \max$ with repeated sprints [26]) intensities. There is an urgent need for future studies to utilise higher exercise intensities (e.g. > 80% ${\dot{\text{V}}\text{O}}_{2} \max$) to replicate racing intensities and, in doing so, more conclusively assess the potential ergogenic effects of adding sodium alginate and pectin to a CHO beverage consumed prior to and during endurance exercise.

### Substrate Oxidation

Initial, mechanistic studies have demonstrated that the pathway of CHO ingestion/intestinal absorption may differ from that of a “standard” CHO beverage when compared with a CHO beverage containing sodium alginate and pectin (i.e. formation of hydrogel in the stomach and faster early GE rate) [11, 12]. However, there has been little evidence of physiological variables relevant to performance being altered. Across the seven studies included within the meta-analysis, only one study demonstrated a difference in the rate of whole-body CHO or fat oxidation, across a wide range of exercise intensities and ingestion rates employed [31].

Regarding whole-body CHO oxidation rates, Achten et al. found significant differences when comparing different types of CHO such as isomaltulose and sucrose [36]. Specifically, isomaltulose ingestion, which is hydrolysed in the intestine at a much slower rate than sucrose [37], results in a significantly lower whole-body CHO oxidation rate and a ~ 50% lower ExCHO oxidation rate. These authors suggest that the low rate of intestinal absorption of isomaltulose was the cause of this dramatically reduced CHO oxidation rate [36]. This study demonstrates the potential of altered absorption rates from the intestine to affect whole-body CHO oxidation rates during exercise; however, the lack of any differences in substrate oxidation with the ingestion of CHO beverages containing sodium alginate and pectin suggests that the hydrogel formed once ingested does not inhibit the hydrolysis of maltodextrin or the intestinal absorption of glucose or fructose, as has been previously theorised [12]. In fact, in direct contention with this theory, is the recent evidence demonstrating higher whole-body CHO oxidation, exogenous CHO oxidation and subsequent performance following the ingestion of 90 g hr^−1^ of CHO with additional sodium alginate and pectin [31].

Despite showing no significant differences in fat oxidation between a CHO beverage or one containing sodium alginate and pectin, Barber et al. highlighted that the ingestion of sodium alginate and pectin was associated with a significant increase in the contribution of fat oxidation to total energy expenditure, resulting in a reduced endogenous CHO utilisation [28]. Once the difference in baseline blood non-esterified fatty acid concentration was included within the analysis as a covariate, differences no longer remained significant, suggesting that the higher fat oxidation was a result of acute differences in the athletes’ baseline characteristics, not due to influence from the sodium alginate and pectin ingestion.

The ExCHO oxidation rate resulting from the ingestion of CHO with the addition of sodium alginate and pectin has been investigated by several authors [27, 28, 30, 31, 33], with the contrasting results mixed for those comparing to an isocaloric control [28, 30, 31], with two finding no differences [28, 30] and one demonstrating a higher ExCHO with the ingestion of CHO with sodium alginate and pectin [31]. The cause of this discrepancy is not clear, but may pertain to the higher exercise intensity resulting in a higher ExCHO [31]. Maximising ExCHO oxidation rate during exercise is well known to be beneficial to endurance performance, and therefore further research determining if the addition of sodium alginate and pectin enhances ExCHO at a range of exercise intensities is required.

### Blood Metabolites

The maintenance of blood glucose concentration is considered one of the major ergogenic features of CHO ingestion during prolonged exercise. The ingestion of CHO with the addition of sodium alginate and pectin has, in line with the other physiological variables measured, been shown by this meta-analysis to have no impact upon blood glucose concentrations during exercise. This finding is unsurprising considering the “downstream” variables that are directly influenced by blood glucose concentration (i.e. performance and substrate oxidation) which were found to be unaffected by the ingestion of a CHO beverage with additional sodium alginate and pectin [24–26, 28–30]. Recently, a pilot study assessed the effects of a CHO beverage containing cross-linked hydroxypropyl cellulose microparticles and sodium alginate compared with an isocaloric control on performance and blood glucose absorption kinetics [32]. A single athlete ran to exhaustion at ~ 60% ${\dot{\text{V}}\text{O}}_{2} \max$ while either ingesting a CHO beverage containing microparticles, sodium alginate and 39 g of glucose or a commercially available sports drink containing the same quantity of glucose. It was found that the glucose absorption kinetics were superior to the ingestion of the CHO beverage containing microparticles and sodium alginate and pectin compared with the control as well as a longer time to exhaustion (~ 2 h 45 min vs ~ 2 h, respectively). While only initial data are available for this study, it suggests that the addition of compounds which may alter the intestinal sensing of CHO and/or alter the intestinal absorption of CHO may have an ergogenic effect on performance, something that has been demonstrated recently [31].

### GID

Initial reports into the use of sodium alginate and pectin to enhance CHO beverage ingestion have suggested that it may reduce the occurrence of GID through differential sensing of CHO within the intestine [10]. Nonetheless, the empirical evidence collected over the last ~ 3 years is less convincing. Each study used within the current systematic review and meta-analysis also investigated potential differences in the occurrence of GID [24–26, 28–30], and each demonstrated that the addition of sodium alginate and pectin did not influence the occurrence of GID. Differences in collection methods, ingestion rates and exercise protocols make the comparison of GID difficult, and therefore GID was not included within the current meta-analysis. That said, important conclusions can be made using the current literature on the influence of sodium alginate and pectin on GID. The most obvious conclusion is that across the range of CHO intake rates (66–90 g hr^−1^), exercise intensities (45–71% ${\dot{\text{V}}\text{O}}_{2} \max$), exercise duration (98–180 min) and exercise modality (cycling or running), the addition of sodium alginate and pectin did not alter the GID response of the participants [24–26, 28–30]. However, to conclude that the addition of sodium alginate and pectin is not beneficial for reducing GID experienced by participants during exercise, as has been done multiple times [17, 24, 26, 28], is not supported by the literature. This conclusion has been made on the evidence that there were no significant differences in GID between a CHO beverage with or without additional sodium alginate and pectin. What is mentioned but not discussed within these studies is that the occurrence of GID within the control beverage is low, which could conceal a beneficial effect in subjects who suffer from severe GID when consuming CHO beverages. For example, the occurrence of GID symptoms was described by Baur et al., as within the “mild range or below” [26], only two participants experiencing “severe” symptoms in a study by McCubbin et al. [24] or the low GID ratings with the control CHO beverage ingestion in the study of Barber et al. [28]. Due to these low incidences of GID associated with the ingestion of the control beverage, it cannot be determined if there was any beneficial effect of sodium alginate and pectin. Notably, however, in two studies, it was found that the ingestion of the CHO beverage containing sodium alginate and pectin resulted in a significantly higher rating of stomach fullness [25, 29]. Due to the gelation process that occurs when the sodium alginate enters the low pH environment of the stomach [11], the viscosity of the ingested beverages increases. This higher sensation of fullness is most likely due to the increase in viscosity associated with sodium alginate ingestion was also demonstrated in a previous study where sodium alginate was ingested with a milk-based meal replacement compared with a control beverage [38]. However, the concentration of alginate in this study [38] was much higher than the studies included within this systematic review (i.e. 1% vs ~ 0.1% alginate by weight, respectively).

## Conclusions

This systematic review and meta-analysis have demonstrated that the current literature shows that there are no significant differences between CHO beverages with or without sodium alginate and pectin when considering performance, substrate oxidation or blood glucose concentration during low (i.e. 45% ${\dot{\text{V}}\text{O}}_{2} \max$) to moderate (i.e. ~ 70% ${\dot{\text{V}}\text{O}}_{2} \max$) exercise intensities. A limitation of the currently published research is the lack of studies employing high-intensity exercise, like those experienced during races (i.e. 80–85% ${\dot{\text{V}}\text{O}}_{2} \max$). Additionally, the lack of GID symptoms present in the control beverages makes it difficult to draw conclusions about the potential of sodium alginate and pectin to alleviate GID. Future research is needed to quantify any potential effect of this CHO technology in “real” race situations at high exercise intensities (i.e. > 80% ${\dot{\text{V}}\text{O}}_{2} \max$) with a particular focus on those participants who are known to suffer from GID when ingesting CHO.

## Data Availability

All data used within this manuscript are available upon request.
